# Targeting regulatory T cells for immunotherapy in melanoma

**DOI:** 10.1186/s43556-021-00038-z

**Published:** 2021-04-19

**Authors:** Lili Huang, Yeye Guo, Shujing Liu, Huaishan Wang, Jinjin Zhu, Lingling Ou, Xiaowei Xu

**Affiliations:** Department of Pathology and Laboratory Medicine, Perelman School of Medicine, University of Pennsylvania, Philadelphia, PA 19104 USA

**Keywords:** Tregs, Melanoma, Immunotherapy, Immune suppression

## Abstract

Regulatory T cells (Tregs) are essential in the maintenance of immunity, and they are also a key to immune suppressive microenvironment in solid tumors. Many studies have revealed the biology of Tregs in various human pathologies. Here we review recent understandings of the immunophenotypes and suppressive functions of Tregs in melanoma, including Treg recruitment and expansion in a tumor. Tregs are frequently accumulated in melanoma and the ratio of CD8^+^ T cells versus Tregs in the melanoma is predictive for patient survival. Hence, depletion of Tregs is a promising strategy for the enhancement of anti-melanoma immunity. Many recent studies are aimed to target Tregs in melanoma. Distinguishing Tregs from other immune cells and understanding the function of different subsets of Tregs may contribute to better therapeutic efficacy. Depletion of functional Tregs from the tumor microenvironment has been tested to induce clinically relevant immune responses against melanomas. However, the lack of Treg specific therapeutic antibodies or Treg specific depleting strategies is a big hurdle that is yet to be overcome. Additional studies to fine-tune currently available therapies and more agents that specifically and selectively target tumor infiltrating Tregs in melanoma are urgently needed.

## Introduction

Regulatory T cells (Tregs) are well known to be involved in the immune regulatory activity [1]. Numerous mechanisms of Tregs mediated immune suppression have been investigated in autoimmune diseases, allergy, acute and chronic infection, pregnancy, and cancer [2, 3]. Depletion of Tregs in systemic immunity evokes severe human disease [4]. With the development of technology, immunophenotypes and functions of Tregs in the tumor microenvironment (TME) have been explored [5]. Tregs are identified as an immune suppressor and they have a crucial role in immune suppression in the TME [6]. The relationship of Tregs with other immune cells in the TME provides strategies to target Tregs in the tumor sites [7]. Treg depletion in tumor tissues promotes anti-tumor activity but may also induce fatal immunotherapy-related adverse events [8]. Despite rapid advances in the preclinical and clinical studies, it is still challenging to fine-tune Tregs to promote anti-tumor responses. Here, we review the biological characteristics and immunologic functions of Tregs in the context of immunotherapy. By understanding the role of Tregs in the TME, new therapies targeting Tregs in melanoma may be developed.

## Phenotypes and functions of Tregs

### Phenotypes of Tregs

Recent studies have established mechanisms for Tregs that mediate immune suppression towards self or non-self-antigens in immunity. In the 1970s, Gershon and colleagues made a breakthrough discovery that a subgroup of T cells dampened immune reactions, and these cells were different from helper T (Th) cells [9]. Subsequently, immunosuppressive cytokines interleukin 10 (IL-10) and transforming growth factor β (TGF-β) were identified in these suppressive T cells [10, 11]. In the 1990s, Sakaguchi et al., defined the suppressive T cells as Tregs through determining the CD25 molecule (the interleukin 2 (IL-2) receptor a-chain) on T cells [12]. CD25^+^ T cells constituted 5% to 50% of CD4^+^ T cells in the human peripheral blood and exhibited immune-suppressive activity [12, 13]. Moreover, the forkhead box P3 (FOXP3) gene was identified as a regulatory gene in CD25^+^CD4^+^ Tregs, which was crucial in autoimmune disease [14]. Therefore, Tregs can be distinguished as CD4^+^CD25^+^FOXP3^+^ T cells in both humans and mice. Recent studies have investigated the different phenotypes and functions of Tregs in the autoimmune diseases with the developmental marker of naive T cells, CD45RA [15]. Tregs are divided into CD45RA^+^FOXP3^lo^CD25^lo^ naive Tregs, CD45RA^−^FOXP3^hi^CD25^hi^ effector Tregs, and CD45RA^−^FOXP3^lo^CD25^lo^ non-Tregs [16]. Naive Tregs derive from the thymus and exhibit weak suppressive activity [7]. These Tregs proliferate and differentiate into effector Treg (eTreg) cells after T cell receptor (TCR) stimulation in the lymph node, acquiring highly suppressive function [17]. The terminally differentiated effector Tregs possess highly suppressive activity but they tend to undergo apoptosis with aging, which contributes to increase reactivity to self-antigens in aging adults [4]. The heterogeneous population of CD45RA^−^FOXP3^lo^CD25^lo^ non-Tregs exhibits no suppressive activity for conventional T (Tconv) cells [18]. Other molecular markers have been suggested to classify Tregs, such as CD127 (IL-7 receptor α), cytotoxic T-lymphocyte-associated protein 4 (CTLA-4), glucocorticoid-induced TNF receptor (GITR), and lymphocyte activation gene-3 (LAG-3). In humans, CD127 is introduced as a new marker for Tregs [19, 20]. It has been suggested that CD4^+^CD25^+^T cells can differentiate into CD127^low^ Tregs and CD127^high^ conventional Th cells [19, 20]. However, researchers have demonstrated that CD127 expression can be downregulated after activation [21, 22]. Therefore, CD127 is identified as a variable marker in Tregs. Several reports have demonstrated that CD4^+^CD25^high^ T cells express CTLA-4, which is correlated with a highly suppressive effect in humans [23, 24]. Similarly, different tissues and interindividual variations seem to determine the expression of CTLA-4 on Tregs [25]. Tumor infiltrating Tregs express much higher CTLA-4 than other T cells. Tanaka and colleagues have classified the human peripheral FOXP3^+^CD4^+^ T cells into three subsets by the expression of various molecules including CTLA-4 (Naive Treg: FOXP3^+^CD25^+^CD45RA^+^CTLA4^+^, Effector Treg: FOXP3^++^CD25^++^CD45RA^−^CTLA4^++^, Non Treg: FOXP3^+^CD25^+^CD45RA^−^CTLA4^+^) as summarized in Table 1. The effector Tregs are highly activated and proliferative with high CTLA-4 expression (FOXP3^++^CD25^++^CD45RA^−^CTLA4^+++^) in melanoma [26, 27]. Similarly, other markers such as GITR and LAG-3 are also used as Treg markers [28, 29]. Higher GITR expression is a marker for Tregs. However, the activity of GITR in the Tregs is controversial. GITR expression not only stimulates Treg expansion but also inhibits Treg suppression [30, 31]. LAG-3 is crucial for the regulation of CD4^+^CD25^+^FOXP3^+^ Tregs which interact with major histocompatibility complex class II (MHC II) molecules on dendritic cells (DCs) [32]. LAG-3 is a phenotypic marker of IL-10-producing Tregs both in vitro and in vivo [33].

**Table 1:** Characteristic of human FOXP3^+^CD4^+^ T cells in blood and tumor

Subsets	Phenotype markers	Characteristics
None Treg	CD45RA^−^ CD4^+^CD25^+^FOXP3^low^ CTLA-4^+^PD-1^+^	No suppressive activity
Naive Treg	CD45RA^+^ CD4^+^CD25^+^FOXP3^low^ CTLA-4^low^ PD-1^−^	Weak suppressive activity Differentiate into effector Treg
Effector Treg	CD45RA^−^ CD4^+^CD25^++^FOXP3^++^ CTLA-4^++^PD-1^+^ GITR^+^LAG3^+^CD127^−^	Strong suppressive activity Prone to apoptosis
Tumor effector Treg	CD45RA^−^ CD4^+^CD25^++^FOXP3^++^ CTLA-4^+++^PD-1^++^ GITR^++^LAG3^++^CD127^−^	High activation and proliferation

### Differentiation and proliferation of Tregs

It is well known that thymus is essential for Treg development. In newborn animals, multiple types of Tregs in lymphoid organs are derived from and developed in the thymus are called thymic Treg (tTreg) cells [4]. A proportion of Tregs is called peripheral Treg (pTreg) cells that develop in the peripheral organs such as intestine after naive T cells exposure to non-self-molecules antigens [34]. However, no reliable markers and methods can completely distinguish the development routes of Tregs. With the development and proliferation, Tregs migrate to lymphoid organs and other tissues via blood and lymphatic ducts with various functions. In physiological conditions, Tregs colonize in lymphoid organs, becoming central Treg (cTreg) cells and non-lymphoid organs such as bone marrow resident effector Treg cells [2]. In response to inflammatory stimulation, for example, acute tissue injury, Tregs expand and recruit to the local injured tissues exhibiting immune suppression [35]. In pathological conditions, cTreg cells convert into effector Tregs, and pTreg cells expand and develop into effector Tregs locally. These Tregs recover the damage tissues by releasing tissue-repairing molecules [36]. However, when the damage is incapable of being repaired completely, for instance, cancer, the Treg pool continues to expand and more Tregs are recruited into the tumor tissue, promoting tumor growth. In cutaneous melanoma, specific chemokine receptors expressed on melanoma cells attract Tregs migrating into tumor sites [37]. Tregs are recruited into tumor tissue by their expressing chemokine receptors such as CC chemokine receptor (CCR) 4, CCR7, CXC-chemokine receptor (CXCR) 4, sphingosin-1-phosphate receptor-1 (S1PR1), and others, which exacerbate the immunosuppressive TME [38–41]. Several cytokines are involved in the differentiation and proliferation of Tregs [42]. Both pTreg and tTreg are characterized by high expression of IL-2α, supporting that IL-2 plays a vital role in the Tregs differentiation [43]. Other common γ-chain cytokines such as interleukin 7 (IL-7) and interleukin 15 (IL-15) may replace the function of IL-2 in the development of tTreg cells [44]. Both IL-2 and IL-15 promote the differentiation of Tregs from tTreg cells [45]. TGF-β is known as a vital growth factor in the peripheral Treg cell differentiation [42].

### Functions of Tregs

Tregs prevent autoimmunity and tissue damage via their immunosuppressive function. Tregs and effector T (Teff) cells mediate self-tolerance and deleterious immune responses against specific self and foreign antigens through mutual regulation [6]. The antigen-specific Teff cells can clear infectious agents, while uncontrolled or inappropriate Teff response results in inflammatory diseases or autoimmune diseases [46, 47]. In contrast, Tregs can prevent the pro-inflammatory and autoimmune response of Teff cells [48, 49]. The balance of tumor-antigen-specific Tregs and Teff cells is crucial for anti-tumor immunity [50, 51]. Reduced Teff: Treg ratios and enhanced Treg suppression have been observed in many cancers. Vaccines containing both CD4 and CD8 epitopes promote Teff: Treg ratios, increase tumor infiltrating lymphocytes and inhibit tumor growth [52]. Tregs also secrete a wide range of soluble anti-inflammatory mediators, such as IL-10, TGF-β, and interleukin 35 (IL-35), which contribute to suppress the proliferation of Tconv cells [53]. Studies have shown that granzyme B derived from Tregs induces effector T cells apoptosis [54, 55]. Loebbermann and co-workers have demonstrated that Tregs can control lung inflammation by expressing granzyme B during acute viral infection [56]. Moreover, Tregs express high CD25 (IL-2 receptor) that forms high affinity with IL-2 and inhibits the proliferation of effector T cells [57]. Thus, Tregs can inhibit effector T cell activation via consumption IL-2. In return, IL-2 produced by Tconv cells stimulates Treg cell expansion and enhances immune suppression [58]. CTLA-4 is a crucial molecule for Tregs in immune suppression [59]. Tregs express high level of CTLA-4 protein, which binds to CD80/CD86 on DCs, weakening their affinity of co-activation of effector T cells [60]. Furthermore, Tregs increase expression of indoleamine 2,3-dioxygenase (IDO) in DCs, which degrades tryptophan into kynurenine [7]. As a result, effector T cells exhibit a stagnation of proliferation, reducing the autoimmunity. In addition, Tregs also use other inhibitory molecules, such as CD39 and CD73 [61], which inhibit the expansion of effector T cells and antigen-presenting cells (APCs) through facilitating extracellular adenosine triphosphate (ATP) conversion into adenosine [2]. Thus, Tregs are involved in various mechanisms of immunosuppression. However, tumor infiltrating Tregs prevent anti-tumor immune response, which differs from their role in suppression of autoimmunity.

## Tregs in the melanoma tumor microenvironment

### Recruitment of Tregs to the tumor microenvironment

**Fig. 1:**
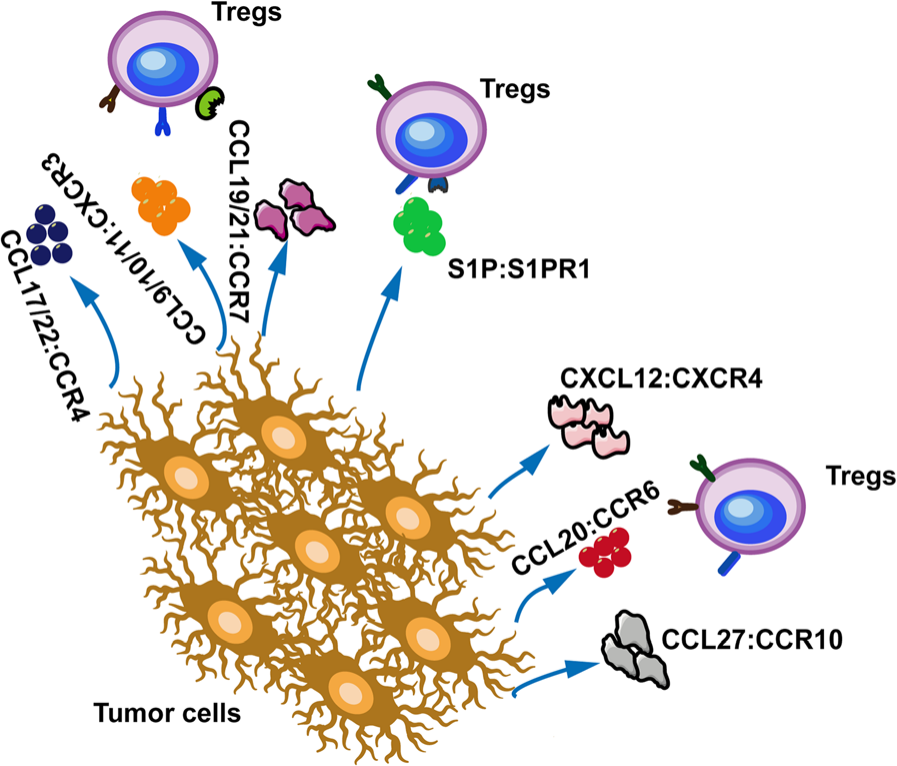
Recruitment of Tregs to the TME. Tregs are recruited to the TME through specific chemokines and their receptors. A number of chemokines are involved in Treg-trafficking to melanoma, including CCL27-CCR10, CXCL12-CXCR4, CCL20-CCR6, CCL19/CCL21-CCR7, CCL9/10/11-CXCR3, CCL17/22-CCR4, and S1P-S1PR1

Naive Tregs (nTregs) homing to the TME is a crucial step for tumor progression [62]. Many chemokines produced by tumor and immune cells drive nTregs into the TME [63]. Studies of tumor infiltrating Tregs of cancer mouse models have demonstrated that specific chemokines and their receptors regulate the preferential homeostasis [64, 65]. A number of chemokines and chemokine receptors are involved in Treg-mediated homeostasis in melanoma, including chemokine (C-C motif) ligand (CCL)27-CCR10, CXCL12-CXCR4, CCL20-CCR6, CCL19/CCL21-CCR7, CCL9/10/11-CXCR3, CCL17/22-CCR4 and S1P-S1PR1 (Fig. 1) [39, 40, 66–69]. CCL17/22-CCR4 are involved in tumor-infiltrating Treg homeostasis and expansion in gastric cancer [64]. Mechanisms of Treg homing to the TME are underexplored. S1P is a bioactive mediator involving in tumor associated Treg expansion, tumor progression, and metastasis [70, 71]. The specific processes include endothelial adhesion, angiogenesis, and cell-cell contact [72]. Effects of S1P in tumor immunity are regulated by its binding to G-protein-coupled receptors S1PR1–5 [73]. Chemokine CXCL9 activates the expression of S1PR1 and S1PR4 on T cells, which induces T cells to migrate from the blood into tissues [74]. Moreover, S1PR1 is known to regulate the proliferation and function of Tregs through mTOR pathway [75]. Furthermore, S1PR1 signaling can activate Tregs and promote Tregs accumulation via signal transducer and activator of transcription 3 (STAT3) pathways and inhibit CD8+ T cells migration [41]. Thus, the S1P-S1PR1 axis plays an essential role in the recruitment of Tregs in melanoma. Effects of other chemokines and chemokine receptors such as CCL27-CCR10 have also been studied in the recruitment of Tregs in melanoma [76, 77].

### Tumor and Treg cell contact mechanisms

**Fig. 2:**
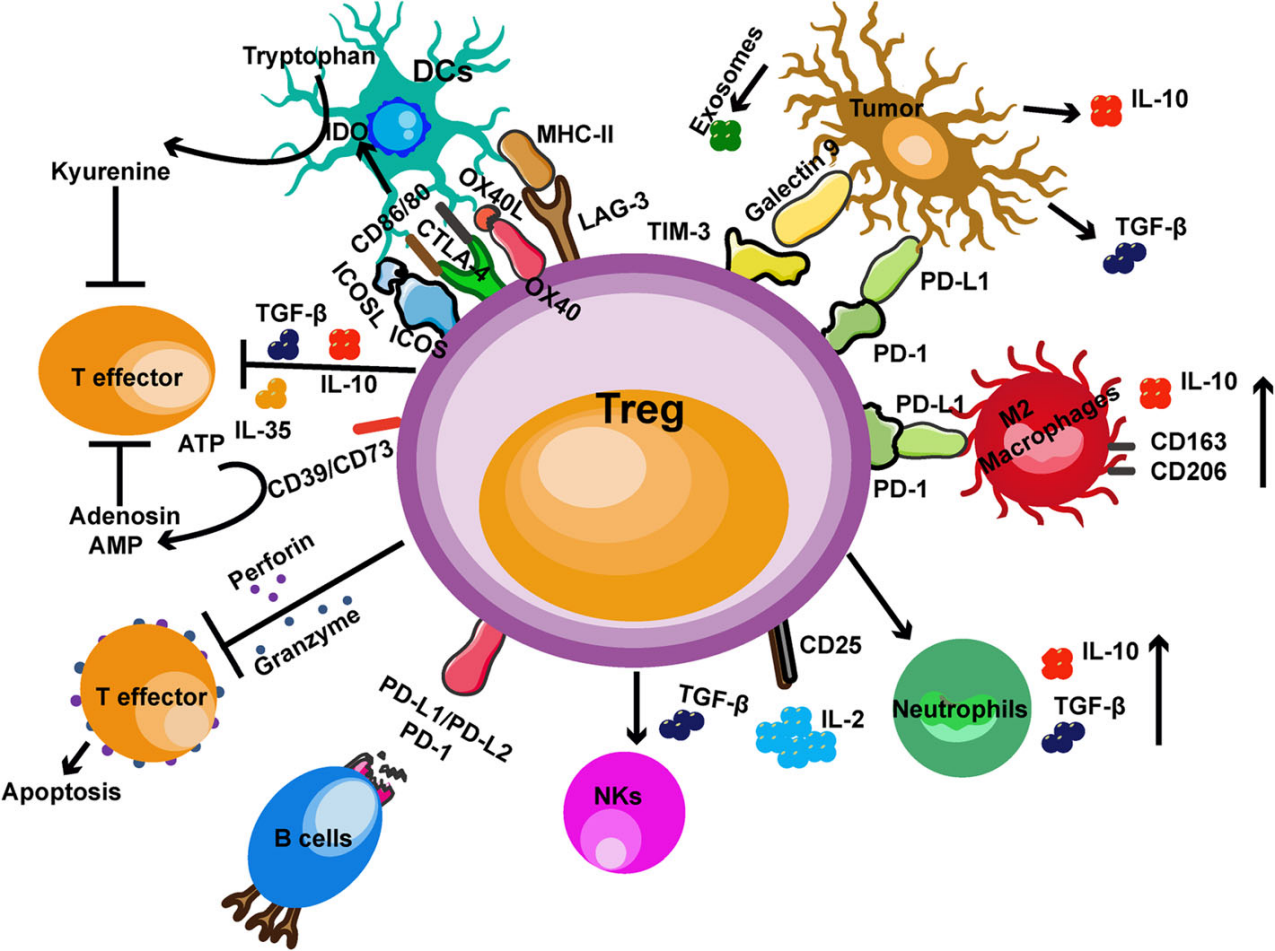
Immune suppressive functions of Tregs in the TME. Tumor cells can produce soluble factors (IL-10 and TGF-β), exosomes, and biological-competent proteins (Galectin 9 and PD-L1), which activate Tregs in the TME. Tregs release anti-inflammatory cytokines (IL-10, IL-35, and TGF-β) that directly inhibit the proliferation of effector T cells and neutrophils. They also produce perforin and granzymes to damage cell membrane and induce T cell apoptosis. In addition, high expression of CD39 and CD73 on Tregs facilitates the conversion of extracellular ATP to adenosine reducing the expansion of effector T cells. Moreover, Tregs consume IL-2 by expressing high level of CD25 (IL-2 receptor) and inhibit the proliferation of effector T cells. Tregs have been suggested to directly inhibit B cell, M2 macrophage, and effector T cells via the PD-1/PD-L1 pathway. Furthermore, Tregs contact with DCs through CTLA-4 and LAG-3. Blocking CTLA-4 can decrease the CD86/80 expression leading to upregulation of IDO. Tregs decrease the proliferation and effector functions of NK cells through IL-2 starvation and TGF-β dependent manner

Various immunophenotypes and functions of Tregs in the TME are associated with the tumor burden [78]. Tumor cells produce soluble factors, extracellular vesicles, and biological-competent proteins, which activate and reprogram Tregs continuously in the TME (Fig. 2) [79–81]. During cancer progression, tumor cells active the TGF-β pathway to mediate tumor growth, invasion, and metastasis [82]. TGF-β is a crucial mediator for FOXP3 in tumor infiltrating Tregs. Ectopic FOXP3 is identified to confer Treg cell suppressive activity; the molecules mediating FOXP3 expression may well regulate the phenotype and function of Tregs [5]. Tumor cells secrete high levels of TGF-β binding to the TGF-β receptor on the Tregs, promoting Treg differentiation and maintenance [83]. Anti-CTLA-4-TGF-βRII fused antibodies significantly reduce the numbers of Tregs and increase CD8+ T cells in a melanoma mouse model [84]. Tumor-associated TGF-β enhances the expansion of Tregs and their immune suppressive function in the TME. IL-10 in the TME is derived from several components including tumor cells. IL-10 mRNA transcripts can be isolated from tumor tissues including ovarian, breast, renal, lung, and skin cancer [85]. Interestingly, IL-10 was originally demonstrated as an important factor in T-cell growth and differentiation [86]. Subsequently, Hsu and colleagues demonstrated that IL- 10 promotes human Treg proliferation through STAT3 and Foxo1 [53]. Moreover, tumor cells-derived exosomes are essential in the intercellular connecting system in the TME. These exosomes express high levels of immunoreceptors and ligands [87]. Exosomes deliver signals from tumor cells to immune cells including Tregs as a mimic profile of tumor cells [88]. Tumor-derived exosomes have been confirmed promoting expansion and immunosuppression of Tregs in the TME [89]. Tregs in the TME are particularly sensitive to exosomes, while CD8+ T cells are mostly inhibited by these exosomes in vitro co-incubation experiments [61]. When tumor-delivered exosomes are in touch with immune cells, they carry IL-10 and TGF-β that enhance Treg expansion and increase expression of CTLA-4 on Tregs to promote immune suppression in melanoma [90]. Melanoma cell-derived exosomes express CD39 and CD73, which significantly enhance Tregs excretion of immunosuppressive adenosine [91]. Tumor-derived exosomes regulate the expression of immune checkpoint proteins that promote the immune suppression of Tregs [92]. Melanoma cells induce Tregs to overexpress various inhibitory checkpoint receptors, including programmed cell death protein 1 (PD-1), CTLA-4, T cell immunoglobulin and mucin domain-containing protein 3 (TIM-3), LAG-3, and T cell immunoglobulin and ITIM domain (TIGIT) [93–96]. Galectin-9 expressed in melanoma cells binds to TIM-3 on Tregs, promoting tumor progression in the mouse or human melanoma tissues [97, 98]. PD-1/programmed death-ligand 1(PD-L1)-programmed death-ligand 2 (PD-L2) axis plays a crucial role in the induction and maintenance of Tregs in the TME [99]. PD-L1 and PD-L2 are highly expressed by tumor cells in the TME and they impair the infiltrating T cell function when binding with PD-1 receptor [100, 101]. It has been shown that there is a profound defect in conversion of naive CD4+ T cells into FOXP3^+^ iTreg cells in the absence of PD-L1 [100]. In addition, PD-L1 converts naive Tregs into effector Tregs by reducing signaling of the AKT–mTOR pathway in naive T cells [100]. Thus, the PD-1/PD-L1-PD-L2 axis synergizes with TGF-β to promote Treg differentiation and maintenance [100].

### Treg and other immune cell contact mechanisms

Tregs exhibit their immune-suppressive effect by multiple mechanisms through production of suppressive soluble cytokines (IL-10, TGF-β, and IL-35), upregulation checkpoint inhibitory receptors (CTLA-4, PD-1, LAG-3, TIM-3, and TIGIT), secretion of granzymes and perforin, and depletion of ATP in the TME [27, 102–105]. Tregs exert different immunosuppression by direct and indirect cell-cell contact mechanisms (Fig. 2) [106]. Tregs produce immunosuppressive cytokines to inhibit effector T cell expansion and release cell membrane soluble mediators, granzymes and perforin, to induce effector T cell apoptosis [107–109]. Tregs also consume IL-2 by expressing high level of CD25 (IL-2 receptor) to inhibit the proliferation of effector T cells [110].

Moreover, high expression of CD39 and CD73 on Tregs facilitates the conversion of extracellular ATP to adenosine, which reduces the expansion of effector T cells and inhibits dendritic and myeloid cells [111]. In melanoma patients, upregulation of CD73 expression was found as a cause for anti-PD-1 therapy resistance, which correlates with poor prognosis [112]. B-cells express high levels of PD-1 and PD-L1 in an antigen-specific manner. Thus, Treg may use PD-1 ligands to directly inhibited B cell activation, suppressed their proliferation via PD-1/PD-L1 axis [113]. Furthermore, Tregs can decrease the proliferation and effector functions of NK cells by IL- 2 starvation in a TGF-β dependent manner [57]. CTLA-4 on Tregs binds to CD80/CD86, and LAG-3 binds to MHC-II, reducing the proliferation of APCs and inducing upregulation of IDO [57], which directly reduces effector T cells and DCs expansion and promotes the differentiation of naive Tregs into effector Tregs [114]. In melanoma patients, Tregs upregulate TIGIT expression and reduce the expression of CD226, which results in a decreased proliferation of effector T cells and DCs [96, 115]. Recently, Tregs was found that they can skew monocyte differentiation into M2 macrophages by reduction of sterol regulatory element-binding protein 1 (SREBP1) in a melanoma mouse model [116]. Other mechanisms of how Tregs affect other immune cells, such as gamma delta T (γδT) cells in melanoma are being investigated.

## Immunotherapy targeting Tregs in melanoma

### Blocking recruitment of Tregs to the TME

**Fig. 3:**
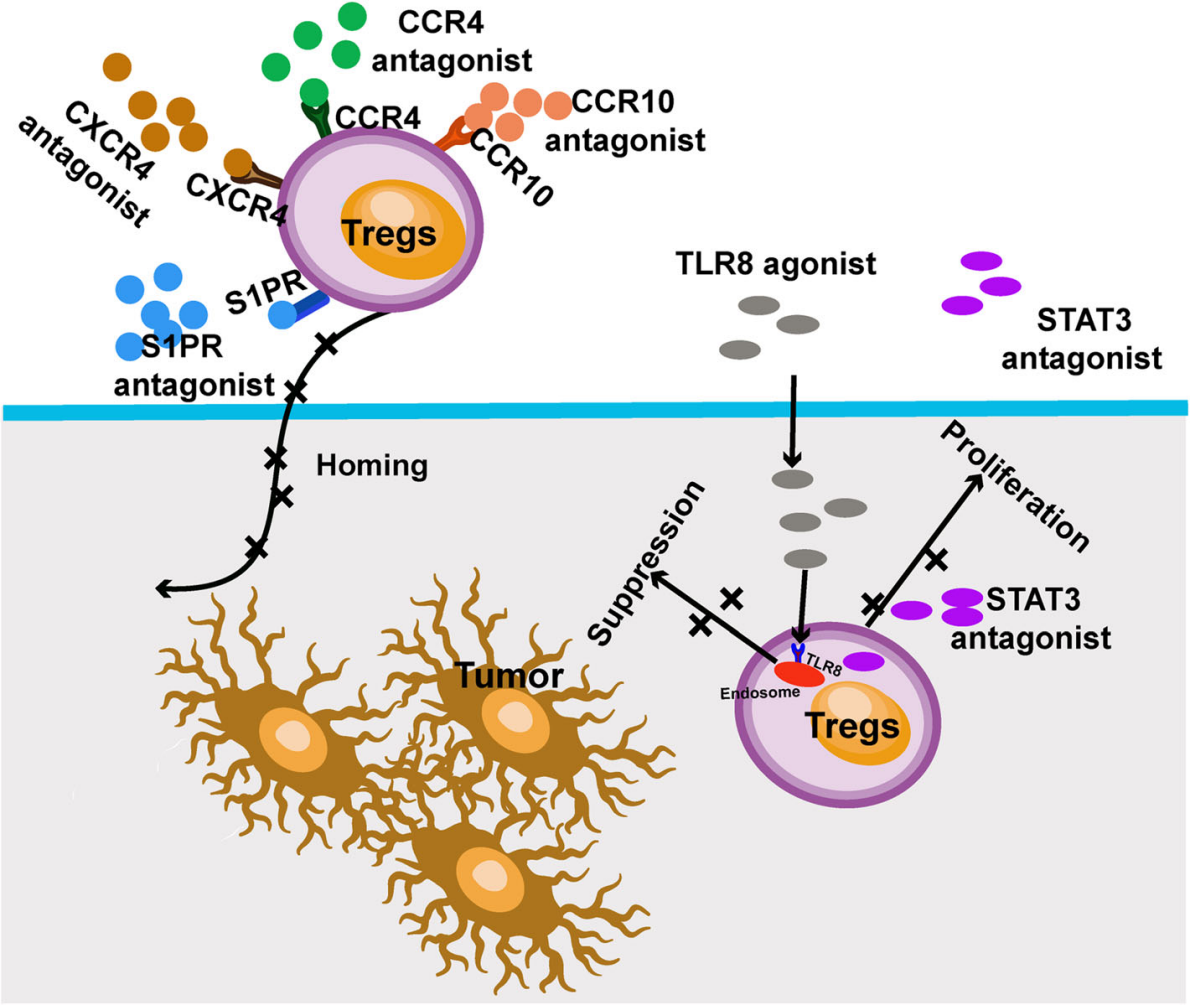
Chemokine and small molecule inhibitors targeting Tregs in melanoma. Anti-CCR4 antibody, CXCR4 antagonist, CCR10 antagonist, and S1PRs antagonist may inhibit recruitment of Tregs to the TME. Other small molecule inhibitors such as STAT3 inhibitors and TLR8 agonists inhibit proliferation of Tregs and enhance TCR activation

As discussed above, many chemokines and chemokine receptors are involved in recruiting Tregs into tumor tissues. Therapies are being explored to prevent Treg recruitment to tumor sites (Fig. 3). CCR4 is expressed on effector Tregs that significantly reduce the immune response in the TME [117]. CCR4 plays a crucial role in Tregs migration and infiltration into tumor site [6]. Moreover, CCR4 is overexpressed in melanoma tissues from brain metastasis compared with primary melanoma [38]. The recruitment by CCR4 can be abrogated by an anti-CCL17 antibody, which blocks CCL17 binding to CCR4 in the TME [118]. A CCR4 antagonist, FLX475, has shown to effectively inhibit Treg migration into the TME and deplete effector Tregs in many tumor models [119]. Clinical trials of combination of FLX475 with pembrolizumab are ongoing for advanced cancers. In melanoma patients, the anti-CCR4 antibody, KM2160, effectively depletes effector Tregs and promotes the immune response of CD8+ T cells in vivo [120]. Only a minor population of patients who received anti-CCR4 monoclonal antibodies (mAbs) experienced severe immune-related adverse events [121]. In addition, animal models demonstrated that it requires a much longer period and profounder degree for autoimmunity than effective antitumor immunity when drugs are applied in Treg depletion [122, 123]. In addition, Sugiyama and colleagues demonstrated that the residual CCR4− eTreg cells and naive Tregs are sufficient to prevent deleterious autoimmunity when using anti-CCR4 mAb to decrease eTreg cells in the immune system [120]. In melanoma patients, high CCR10 expression is associated with a short survival [40]. In addition, overexpression of CCR10 in the B16 melanoma mouse model resulted in increased tumor size and lymph node metastases [124]. CCR10 antagonist (brintonamide D) demonstrates a potential anti-tumor effect in breast cancer [125]. Melanoma patients with higher CXCR4 expression show poorer overall survival [126]. An oral CXCR4 antagonist, X4P-001, improves the efficacy of checkpoint inhibitor therapy and modulates tumor infiltrating immune cells by disrupting the CXCL12-CXCR4 axis [127]. The combination of X4P-001 with anti-PD-1 therapy is being evaluated in clinical trials in advanced melanoma patients. Furthermore, S1PRs antagonist, FTY720, is an immunomodulatory prodrug [128, 129]. FTY720 decreases the recruitment of CD4+ T cells directly and prevents the differentiation of Th1 T cells into Tregs via targeting S1PR1 [130]. In the B16F10 mouse melanoma model, FTY720 induces immunomodulatory effect by inhibiting the recruitment of Tregs into tumor tissues [131].

### Small molecule antagonists for Treg depletion

STAT3 plays an essential role in tumor progression and tumor immunity [132]. Phosphorylation of STAT3 mediates immune escape and is associated with poor survival in melanoma patients [133]. STAT3 knockout mice have reduced tumor infiltrating Tregs and pronounced anti-tumor response [134]. Therefore, STAT3 is a potential target for melanoma immunotherapy (Fig. 3). WP1066, a novel small molecule inhibitor of STAT3, exhibits significant antitumor effect in advanced melanoma patients by inhibiting the proliferation of Tregs and enhancing the TCR activation on ZAP-70 [135]. Similarly, another small molecule STAT3 inhibitor, Stattic, decreases the immune suppressive function of Tregs in vitro [93]. Another small molecule STAT3 inhibitors, OPB-31121, inhibited cancer cell lines by targeting the STAT3-SH2 domain. However, the clinical trials for these drugs were abrogated due to low anti-tumor activity, high toxicity events, and poor pharmacokinetics [136]. In addition, toll like receptor 8 (TLR8) activation on Tregs can prevent their suppression on effector T cells and DCs [137]. TLR8 reverses immunosuppression by suppressing the glucose uptake and the process of glycolysis in Tregs [138]. These findings need to be confirmed in human studies in the future. Nevertheless, depletion of tumor infiltrating Tregs by signaling molecules is a potential promising therapy for melanoma.

### Immune checkpoint blockade therapies targeting Tregs

**Fig. 4:**
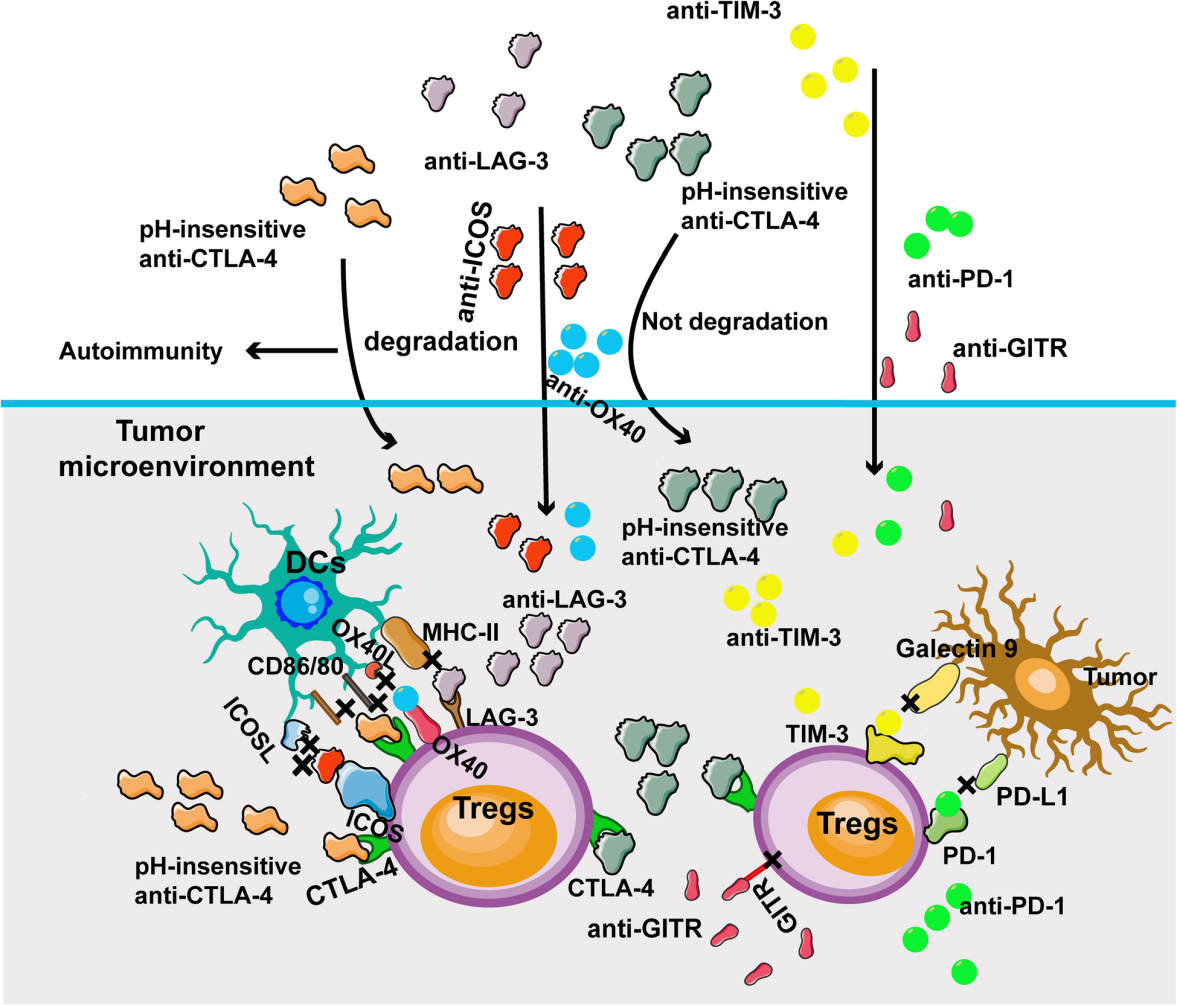
Immune checkpoint therapies targeting Tregs in melanoma. In the TME, anti-PD-1 mAbs block the PD-1 function on the Tregs. Anti-CTLA-4 mAbs induce Treg cell depletion by activating the Fc receptors. However, current CTLA-4 mAbs often result in severe immune-therapy related adverse events. A new version of anti-CTLA-4 (pH-sensitive CTLA-4) mAbs do not degrade in the lysosomal and recirculate to the cell surface of Tregs, reducing the adverse immune events. Anti-TIM-3 mAbs exhibit dual functions in depleting Tregs and stimulating CD8^+^ T cells. Anti-LAG-3 mAbs prevent Treg recruitment and promote DCs function in melanoma therapy. Other antibodies targeting OX-40 and ICOS inhibit proliferation of Tregs in the TME. The anti-GITR mAbs reduce circulating and intratumor Tregs in advanced melanoma patients

The anti-PD-1 antibody is one of the recent breakthroughs in cancer immunotherapy, which has excellent results in many cancers [139, 140]. Nevertheless, only a minority of patients who receive anti-PD-1 therapy exhibits significant responses [141, 142]. Resistance to anti-PD-1 therapy in melanoma correlates with lymphatic vessel density in the tumor tissues and lymph nodes [143]. Blockade of PD-1/PD-L1 axis induces the recruitment of exhausted T cells and Tregs in anti-PD-1 therapy-resistant melanoma patients [144]. The PD-1 expression on Tregs function similarly to that on effector T cells; and the anti-PD-1 mAbs may activate the immunosuppressive function of Tregs in the TME (Fig. 4) [145]. While another study found that anti-PD-1 mAbs , pembrolizumab, did not affect the phenotype or function of Tregs through PD-1/PD-L1 axis [146]. Thus, carefully designed studies to elucidate the effects of anti-PD-1 therapy on Tregs are needed. A few recent studies suggest that anti-CTLA-4 mAbs play a major role in regulating the function of tumor infiltrating Tregs. As discussed above, CTLA-4 is highly expressed on activated Tregs and also upregulated in activated CD4^+^ and CD8^+^ T cells in melanoma tissues compared to other tumors [7, 27]. Anti-CTLA-4 mAbs were initially thought to suppress the inhibition on activated CD4^+^ and CD8^+^ T cells and augment the anti-tumor immune response in the TME [147]. However, more recent studies discover that anti-CTLA-4 mAbs predominantly deplete Tregs in the TME to promote the anti-tumor immune response [148]. Treg depletion by anti-CTLA-4 mAbs decreases the immunosuppression in the TME, but it also results in severe cancer immunotherapy-related adverse events [59]. Thus, maintenance of balanced Tregs in the immune system and the TME is crucial for preventing cancer immunotherapy-related adverse events while promoting anti-tumor response.

TIM-3 is expressed on Tregs with enhanced regulatory function [104]. However, TIM-3 is rarely found on Tregs in the peripheral immune system, the majority of tumor-associated Tregs express TIM-3 comprising a particular subset of tissue Treg [149]. Thus, it is possible to target TIM-3 in the TME in cancer immunotherapy (Fig. 4). In the preclinical mouse melanoma model, blockade TIM-3 exhibits anti-tumor response by stimulating CD8^+^ T cells [150]. Moreover, TIM-3 signaling appears to be a crucial mediator in both innate and adaptive immune responses. Therefore, targeting TIM-3 combined with other checkpoints such as anti-PD-1 mAbs is currently being tested as a new cancer immunotherapy strategy. Recently, a combination of TIM-3 inhibitor TSR-022 and PD-1 inhibitor dostarlimab is conducted at the University of Pittsburgh in a clinical trial for Stage III or IV melanoma patients. Similar to TIM-3, LAG-3 expressed on a variety of T cells, including CD4^+^ T cells, CD8^+^ T cells, and Tregs. LAG-3 expression is essential for Treg cell function and is also associated with treatment resistance [151]. Recent studies have demonstrated that LAG-3 blockade prevents Treg recruitment and promotes DCs function in melanoma treatment [152]. Nevertheless, anti-LAG-3 therapy is still in its infancy, further studies are needed to explore the therapeutic efficacy in various tumors. Furthermore, other molecules are explored to target Tregs either deliberately or inadvertently in cancer immunotherapy such as TIGIT, V-domain Ig suppressor of T cell activation (VISTA), and CD73.

In addition to checkpoint inhibitors, other molecules can also inhibit immune suppressive functions of Tregs. GITR is a member of the tumor necrosis factor receptor family. It is expressed on Tregs and serves as a mediator in Treg regulated immunosuppression [153]. Anti-GITR mAbs abrogate tumor infiltrating Tregs, decrease the suppressive function of Tregs, and promote the effector function of Tconv cells in a preclinical melanoma model [154]. The anti-GITR mAbs (TRX518) reduce circulating and intratumoral Tregs and demonstrate significant clinical efficacy in advanced melanoma patients [155]. In addition, combination of anti-PD-1 mAbs with TRX518 reverses the resistance to anti-GITR therapy in a mouse tumor model [155]. The efficacy of the combination therapy should be explored in patients with advanced melanoma.

OX40 (CD134) is a co-stimulatory molecule on Tregs [156]. Earlier studies on tumor inhibition using anti-OX40 antibody showed that anti-OX40 mAbs augmented anti-tumor immunity with the depletion of tumor infiltrating Tregs in several types of cancer animal models [157, 158]. Moreover, OX40 antibodies, such as MOXR0916, demonstrated excellent therapeutic effect in some patients with low adverse events in a phase I trial [157]. Inducible T-cell costimulator (ICOS) that binds to ICOS ligands on APC has also been identified on the Tregs [159]. Recent studies have shown that increased number of ICOS^+^ Tregs is found in various cancers, including melanoma [160] and breast cancers [161]. Increased proliferation of ICOS^+^ Tregs is also found in melanoma patients after IL-2 therapy [162]. Thus, targeting ICOS on Tregs and interrupt the interaction between ICOS and ICOSL may be an effective measure for anti-tumor immunity. In preclinical studies of an ICOS agonistic mAbs alone showed a promising effect and but resulted in severe side effect [163]. Another ICOS agonistic mAbs (JTX-2011) is currently being tested in a clinical trial alone and in combination with anti-PD-1 mAbs.

### Vaccine immunotherapy for Treg depletion

A potential application of anti-cancer vaccine may be used to target Tregs [164]. Vaccination targeting FOXP3 provides an excellent measure for depleting Tregs in anti-tumor immunity [165]. A study has been performed by using Fox-Fc DNA vaccine/recombinant FOXP3-Fc fusion protein, which demonstrates an increased cytotoxic T lymphocytes (CTL) response against FOXP3 Tregs [166]. Moreover, in the B16 melanoma mouse model, the DC vaccine exhibits a significant antitumor effect by enhancing the CTL response and decreasing the percentages of FOXP3^+^ Tregs [165]. Tumor cell vaccine plus FOXP3 gene silencing inhibits tumor growth and enhances the efficacy of vaccination immunotherapy [167]. Similarly, therapy that combined dendritic cell-based tumor vaccine with toll-like receptor 7 (TLR7) agonist showed excellent anti-tumor response that resulted in a decrease of tumor infiltrating Tregs [168]. However, more studies are needed for vaccine-based therapies against Tregs.

## Challenges of targeting Tregs in melanoma immunotherapy

### Tregs: a friend or foe for melanoma

Based on the immune suppressive role of Tregs, their presence in the TME is expected to be associated with tumor progression and short survival in cancer patients. However, tumor infiltrating Tregs seem to correlate with a favorable overcome in cancers with characteristics of chronic inflammation, such as colorectal cancer [169]. In colorectal cancers, abundant tumor infiltrating naive FOXP3^low^ Tregs exhibit better survival than those patients with FOXP3^hi^ Tregs in the tumors. The differentiation and proliferation of inflammatory FOXP3^low^ naive Tregs are dependent on the production of IL-2 and TGF-β by tissues [170]. Strategies that deplete the FOXP3^hi^ Tregs and increase the FOXP3^low^ naive Tregs in the tumor tissue might show high anti-tumor therapeutic efficacy [170]. Remarkably, genetic depletion of caspase recruitment domain-containing membrane-associated guanylate kinase protein-1 (CARMA1) which is a critical component mediated by TCR engagement in FOXP3^hi^ Tregs produces an anti-tumor effect without affecting systemic autoimmunity [171]. Moreover, combination of anti-PD-1 mAbs with CARMA1 deletion therapy reverses resistance to PD-1 blockade therapy in cancer [171]. By analyzing PBMC from melanoma patients, flow data classifies FOXP3^+^ cells into FOXP3^hi^ Tregs, FOXP3^low^ naive Tregs, and FOXP3^low^ non-Tregs [172]. With tumor progression, both FOXP3^hi^ Tregs and FOXP3^low^ Tregs increase in melanoma patients [172]. Despite methods to suppress different Treg subpopulations in melanoma have not been discovered, targeting subpopulation of Tregs in the TME may be proven as an effective cancer therapy without concomitant significant adverse immunological reactions.

### Balance of autoimmunity and Treg-targeting cancer immunotherapy

As described above, CTLA-4-targeting immunotherapy significantly depletes tumor infiltrating Tregs but may also induce fatal immunotherapy-related adverse events in some patients. Anti-CTLA-4 mAbs suppress the binding of CTLA-4 to CD80 and CD86 [173]. These actions promote the tumor-activated T cells migrating into the tumor site, and enhance the anti-tumor therapeutic effect. The therapeutic effect of CTLA-4 mAbs is determined by their ability to engage Fc receptors for antibody-dependent cell mediated cytotoxicity (ADCC) on host cells [59]. The interaction between CTLA-4 mAbs and the activating Fc receptors is critical for selective depletion of Tregs in the tumor. In addition, the anti-CTLA-4 mAbs selectively reduce Tregs in the tumor sites by activating the Fc receptors, increasing the anti-tumor activity in cancer immunotherapy [148]. To reduce anti-CTLA-4 mAbs associated adverse immune reaction, it is urgent to discover new versions of anti-CTLA-4 mAbs. A pH-sensitive CTLA-4 antibody has been shown to reduce cancer immunotherapy-related adverse events with increased anti-cancer activity in the tumor site [174]. pH-insensitive CTLA-4 mAbs degraded by lysosomal may cause autoimmune events [148]. However, pH-sensitive CTLA-4 mAbs maintain their bioavailability that is not degraded by the lysosomal, recirculate to the Treg surface, reduce adverse immune events, and exert high anti-tumor efficacy in the TME [175]. One of the pH-sensitive CTLA-4 mAbs, ONC-392, has entered in the clinical trials of metastatic melanoma patients. It may be important for patients to perform human leukocyte antigen (HLA) haplotypes analysis to determine susceptibility to autoimmunity in Treg depletion treatment. Intratumor immunotherapy may also resolve the issues of immune-related adverse events caused by the immune checkpoint blockade [176]. Intratumor immunotherapy could selectively abrogate the tumor infiltrating Tregs by not affecting naive Tregs elsewhere. Sato and colleagues demonstrated that tumor-associated Tregs were depleted by near-infrared photoimmunotherapy in a preclinical study [177]. Studies have shown that combination of anti-CTLA-4 with anti-PD-1 mAbs provides an excellent immune response but also causes severe immune-related adverse events [178]. Intratumoral administration of smaller dose of Treg depleting antibodies or immune checkpoint antibodies is a potential promising therapy. However, additional clinical trials are needed to test this approach.

## Conclusions and prospects

Tregs play a crucial role in the melanoma progression. Tumor infiltrating Tregs migrate into the TME via specific chemokines and chemokine receptors to promote tumor growth by enhancing immune suppression. The immune suppressive functions of Tregs in the TME are multifaceted through production of suppressive soluble cytokines (IL-10, TGF-β, and IL-35), upregulation of checkpoint inhibitory receptors (CTLA-4, PD-1, LAG-3, TIM-3, and TIGIT), secretion of granzymes and perforin, consumption of IL-2 and depletion of ATP in the TME. Subpopulations Tregs in the TME may have different functions. By understanding the mechanisms of Treg differentiation, recruitment, expansion, and immune suppression, therapeutic strategies of depleting subpopulation of Tregs can be developed to increase anti-tumor response without causing severe adverse immune response. Targeting tumor infiltrating Tregs may be achieved using antibodies to TIM-3, LAG-3, TIGIT, VISTA, and CD73. CTLA-4-targeting immunotherapy significantly depletes tumor infiltrating Tregs, but it also may induce severe immunotherapy-related adverse events. Cancer immunotherapy that aims at depletion of tumor infiltrating Tregs needs the balance of anti-tumor response and autoimmunity.

## Data Availability

Not applicable.

## Abbreviations

Treg: Regulatory T
IL-10: Interleukin 10
TGF-β: Transforming growth factor β
IL-2: Interleukin 2
FOXP3: Forkhead box P3
Teff: Effector T
Tconv: Conventional T
CTLA-4: Cytotoxic T-lymphocyte-associated protein 4
GITR: Glucocorticoid-induced tumor necrosis factor receptor family-related gene
LAG-3: Lymphocyte activation gene-3
Tfh: T follicular helper cells
Th: T helper cells
DCs: Dendritic cells
NK: Natural killer
MHC-II: Major histocompatibility complex class II
tTreg: Thymic Treg
pTreg: Peripheral Treg
cTreg: Central Treg
eTreg: Effector Treg
CCR: CC chemokine receptor
CXCR: CXC-chemokine receptor
CCL: Chemokine C-C motif ligand
S1PR1: Sphingosin-1-phosphate receptor-1
IL-7: Interleukin 7
IL-15: Interleukin 15
IL-35: Interleukin 35
nTreg: Naive Treg
IDO: Indoleamine 2,3-dioxygenase
APCs: Antigen-presenting cells
TME: Tumor microenvironment
STAT3: Signal transducer and activator of transcription 3
PD-1: Programmed cell death protein 1
TIM-3: T cell immunoglobulin and mucin domain-containing protein 3
TIGIT: T cell immunoglobulin and ITIM domain
PD-L1: Programmed death-ligand 1
PD-L2: Programmed death-ligand 2
ATP: Adenosine triphosphate
SREBP1: Sterol regulatory element-binding protein 1
γδT: Gamma delta T
VISTA: V-domain Ig suppressor of T cell activation
ICOS: Inducible T-cell costimulator
mAbs: Monoclonal antibodies
CTL: Cytotoxic T lymphocytes
OVA: Ovalbumin
TLR7: Toll like receptor 7
TLR8: Toll like receptor 8
CARMA1: Caspase recruitment domain-containing membrane-associated guanylate kinase protein-1
TCR: T cell receptor
ADCC: Antibody-dependent cell mediated cytotoxicity
HLA: Human leukocyte antigen
